# Efficacy of LASER for de-epithelialization of free gingival graft: a systematic review

**DOI:** 10.1007/s10103-025-04554-0

**Published:** 2025-06-27

**Authors:** Parham Hazrati, Sahar Baniameri, Hamoun Sabri, Dumitru Chele, Sandra Stuhr

**Affiliations:** University of Michigan–Ann Arbor, Ann Arbor, USA

**Keywords:** Lasers, Periodontics, Dental implants, Laser therapy, Connective tissue graft, Free gingival graft, De-epithelialization

## Abstract

This systematic review primarily aimed to investigate the efficacy of de-epithelializing free gingival graft (FGG) using LASER and, secondarily, to evaluate the clinical outcomes of soft tissue augmentation using LASER-de-epithelialized connective tissue grafts (CTGs). Following PRISMA guidelines, an electronic search was conducted in PubMed/MEDLINE, Scopus, Embase, and Web of Science databases without date or language restrictions on February 4, 2025. Clinical studies employing LASER for de-epithelializing FGG were considered eligible. The primary outcome was successful epithelium removal, while the main secondary outcomes were mean and complete root coverage percentage. Quality appraisal of the included studies was executed according to the revised Cochrane risk-of-bias tool for randomized trials (RoB2) and Risk of Bias in Non-randomized Studies - of Interventions (ROBINS-I). Of 243 studies, three met all eligibility criteria. Two randomized controlled clinical trials (RCTs), and one pre-post study were included. CO_2_, Er: YAG, and diode LASERs were utilized in the included studies. Power was set to 1 and 2 W in two and one studies, respectively. Epithelium removal was not quantitatively reported in any of the included studies, but complete removal of epithelium with LASER was reported qualitatively in one study. In all the included studies, LASERs yielded clinical outcomes comparable to blades, with minor superiorities in aesthetic outcomes observed with LASERs. LASERs show potential for successful removal of epithelium from FGG without causing safety issues or complications. However, the evidence on this topic remains limited, highlighting the need for well-conducted and reported RCTs.

**Registration** The protocol of this systematic review was registered on PROSPERO with the ID CRD42025646636.

**Clinical Trial Number** Not applicable.

**Supplementary Information:**

The online version contains supplementary material available at 10.1007/s10103-025-04554-0.

## Introduction

The connective tissue graft (CTG) poses significant clinical applications in periodontal practice, including treatment of gingival recession and soft tissue augmentation around teeth and implants [1, 2]. Although a variety of new materials and biologics have been introduced as an alternative to CTGs, evidence shows that the CTG is still the gold standard for these applications and has considerable superiority with regard to clinical attachment loss (CAL) reduction, keratinized tissue width (KTW) gain, recession depth reduction, and complete root coverage (CRC), particularly in the long-term [3, 4]. Nevertheless, CTG has significant disadvantages, such as pain, infection, extensive bleeding, and keloid-like scar formation at the donor site, which is primarily the lateral palate followed by the tuberosity [5, 6].

There are two main approaches for harvesting a CTG from the palate. The first method typically involves creating a flap in the premolar region of the palate. When vertical releasing incisions are present, this technique is referred to as the “trap-door technique” [7, 8]. Conversely, if only a single incision is used, it is known as “single-incision” or “envelope technique” [9, 10]. The CTG obtained using these techniques, which permit repositioning of the palatal flap, is typically referred to as a sub-epithelial connective tissue graft (SCTG). Notably, this approach should be avoided when the palatal fibromucosa lacks sufficient thickness [11]. Alternatively, Zucchelli et al. introduced the de-epithelialized gingival graft (DGG), emphasizing that healing by secondary intention does not necessarily lead to increased post-operative morbidity and discomfort [12]. This technique involves first harvesting a free gingival graft (FGG) and then performing extra-oral de-epithelialization, which refers to complete removal of the epithelium from the graft [12]. A systematic review by Tavelli et al. concluded that using a DGG with a coronally advanced flap (CAF) yields superior clinical outcomes in treating gingival recession compared to a SCTG [13].

A relatively less common complication of CTG is the formation of cyst-like structures in recipient site that usually develops months or years after the surgical procedure. This condition typically manifests as a small opening in the gingiva, from which a thick, white exudate may be released when pressure is applied to the facial aspect of the gingiva [6]. Currently, our understanding of the etiology of this complication is incomplete, mainly due to the scarce number of reported cases [14, 15], but upon histological assessment of removed cyst-like lesions, invaginations and epithelial remnants with the potential to giving rise to this condition have been witnessed [6, 16]. In most cases, epithelial remnants or deep projections of epithelium in CTG have been identified as the main source and etiology of these cysts [6, 14, 15]. Therefore, when harvesting techniques involve a graft with an epithelial layer, it is essential to thoroughly remove the superficial epithelium; however, it has been reported that even with careful removal with a blade, residual epithelium remains in up to 80% of harvested CTGs [17–19].

In order to overcome the limitations of conventional methods of de-epithelializing CTGs, several techniques and devices have been proposed, such as bone scrapers [20], rotary abrasive instruments [21, 22], and ablation with LASERs [23]. Although these techniques have addressed several limitations of conventional de-epithelialization techniques using a blade, each method presents unique advantages and disadvantages. Bone scrapers function analogously to dermatomes used for skin, allowing clinicians to achieve a standardized and uniform removal of epithelium due to the consistent depth provided by the scraper. However, this technique incurs higher costs, as it requires the use of disposable scrapers [20]. Conversely, the use of rotary abrasive instruments, such as reusable diamond burs, can mitigate this cost-related drawback. Additionally, rotary instruments are advantageous for patients with thin palatal soft tissue, as they help preserve connective tissue by minimizing the removal of the lamina propria. Nevertheless, a potential limitation of intraoral epithelialization techniques is the difficulty of access in patients with restricted mouth opening or a hypersensitive gag reflex [19]. Following intraoral de-epithelialization—whether performed using a round diamond bur with a low-speed handpiece or by scraping with a Kirkland knife—epithelial remnants can still be detected. When using a bur, these remnants are generally less extensive than those left after extraoral de-epithelialization with a blade [24], yet they still cover between 4.57% and 29.12% of the surface area [22]. In contrast, intraoral de-epithelialization with a Kirkland knife tends to leave more epithelial tissue compared to extraoral blade techniques [25].

The efficacy of removing or ablating epiderma or epithelium with LASER has been investigated extensively in several disciplines of medicine, including ophthalmology [26], dermatology [27], and urology [28]. Similarly, in periodontics, researchers have employed LASER technology to de-epithelialize FGG [23, 29]. However, to the best of the authors’ knowledge, no comprehensive review article provides an overview of the current techniques, their outcomes, and their efficacy. Therefore, this study aims to primarily assess the efficacy of LASER in de-epithelializing FGG and, secondarily, to evaluate the clinical outcomes of soft tissue augmentation using LASER-de-epithelialized CTG through a comprehensive and systematic review of the available literature.

## Materials and methods

### Study design, protocol, and registration

This research was conceptualized as a systematic review and was conducted in accordance with the latest guidelines of Preferred Reporting Items for Systematic Reviews and Meta-Analysis (PRISMA) 2020 statement (Supplementary Tables 1 and 2) [30]. Also, the study protocol was registered in the International Prospective Register of Systematic Reviews (PROSPERO) portal with the reference number CRD42025646636 prior to study initiation. Institutional Review Board (IRB) approval was not required since this study relied solely on previously published data.

### PICOST elements and focus question

The following PICOST elements were defined to formulate the focused question.

**Population**: Patients (humans) who need one or multiple connective tissue grafts for periodontal or peri-implant soft tissue augmentation.

**Intervention**: De-epithelializing FGG with a LASER, either intra- or extra-orally.

**Comparison**: Other methods of de-epithelialization, including using blade, or none.

**Outcomes**: Primarily, the efficacy of epithelium removal and, secondarily, clinical outcomes of soft tissue augmentation, including CRC, mean root coverage (MRC), KTW gain, recession depth (RD) reduction, among others.

**Study design**: Any human prospective clinical study with equal to or more than 5 patients, such as prospective randomized or non-randomized controlled clinical trials, pre-post studies with a proper protocol, etc.

**Timeframe**: Articles published at any point in time.

Based on the mentioned framework, the following focused question was considered:

In prospective clinical studies with at least 5 patients requiring connective tissue grafting, what is the efficacy of LASER de-epithelialization of FGG, and how does it compare to other methods, including blade de-epithelialization?

### Eligibility criteria

As mentioned in the PICOST framework, any clinical study with equal to or more than 5 patients for whom CTG is indicated will be included. Animal studies, cadaver studies, case reports, conference abstracts, review studies, and letters were not included. Additionally, studies that utilized LASER for graft harvesting or low-level laser therapy (LLLT) of donor or recipient site for managing the symptoms or improving the outcomes were excluded.

### Search strategy

The online databases PubMed/MEDLINE, Scopus, Web of Science, Cochrane Central Register of Controlled Trials (CENTRAL), and Embase were systematically searched using the queries outlined in Table 1. The search was conducted without imposing any restrictions on publication type, language, or year of publication. In addition to reviewing the reference lists of articles deemed eligible for inclusion, issues published after 2000 from of a list of selected journals (Supplementary Table 3) were also manually examined.

**Table 1:** Search queries

Database	Date	Search Query	Result
Web of Science	Feb 4, 2025	TS = ((Laser*) AND (De-epithelial* OR De epithelial* OR Deepithelial* OR Depithelial* OR Decorticat* OR Debrid* OR Denud* OR Epithelium OR Epithelial) AND (((Connective Tissue OR Gingival OR Gingiva OR Mucosal OR Mucosa OR Mucous Membrane OR Palate OR Palatal OR Tuberosity) AND Graft) OR CTG OR FGG OR SCTG))	164
Embase		(Laser*) AND (De-epithelial* OR De epithelial* OR Deepithelial* OR Depithelial* OR Decorticat* OR Debrid* OR Denud* OR Epithelium OR Epithelial) AND (((Connective Tissue OR Gingival OR Gingiva OR Mucosal OR Mucosa OR Mucous Membrane OR Palate OR Palatal OR Tuberosity) AND Graft) OR CTG OR FGG OR SCTG)	114
PubMed/MEDLINE		(“Lasers“[MeSH Terms] OR “Laser Therapy“[MeSH Terms] OR “laser*“[All Fields]) AND (“de-epithelial*“[All Fields] OR “de epithelial*“[All Fields] OR “deepithelial*“[All Fields] OR “depithelial*“[All Fields] OR “decorticat*“[All Fields] OR “debrid*“[All Fields] OR “denud*“[All Fields] OR “Epithelium“[All Fields] OR “Epithelial“[All Fields]) AND (((“Connective Tissue“[All Fields] OR “Gingival“[All Fields] OR “Gingiva“[All Fields] OR “Mucosal“[All Fields] OR “Mucosa“[All Fields] OR “Mucous Membrane“[All Fields] OR “Palate“[All Fields] OR “Palatal“[All Fields] OR “Tuberosity“[All Fields]) AND “Graft“[All Fields]) OR “CTG“[All Fields] OR “FGG“[All Fields] OR “SCTG“[All Fields])	48
Scopus		TITLE-ABS-KEY ((Laser*) AND (*epithelial* OR Decorticat* OR Debrid* OR Denud* OR Epithelium OR Epithelial) AND (((Connective Tissue OR Gingival OR Gingiva OR Mucosal OR Mucosa OR Mucous Membrane OR Palate OR Palatal OR Tuberosity) AND Graft) OR CTG OR FGG OR SCTG))	30

### Study screening and data extraction

The initial screening process involved evaluating the titles and abstracts of the identified studies based on the predefined eligibility criteria in the citation manager software (EndNote V21.5). This step was performed independently by two authors (P.H. and S.B.) to ensure maximum precision in article selection. Following this, the full texts of the selected citations were retrieved for a more comprehensive assessment. This process was conducted independently by two reviewers (P.H. and S.B.), with any discrepancies resolved through discussion with a third expert reviewer (S.S.). Inter-examiner agreement was assessed with Cohen’s kappa statistic for each stage of screening.

Subsequently, two authors (P.H. and H.S.) extracted key data items from the full texts and systematically organized them into predesigned tables. If any of the emission parameters were not reported in the included studies, the official user manual of the LASER device was retrieved and consulted to obtain missing values. In cases of disagreement, a senior author (S.S.) provided resolution to ensure accuracy and consistency in data extraction. Due to the predominantly qualitative and subjective nature of both the data extraction process and the data collected from the included studies, calculation of inter-examiner agreement using kappa statistics was not feasible for this step.

### Risk of bias assessment

Two investigators (P.H. and H.S.) independently assessed the risk of bias in the included studies using the Cochrane risk-of-bias tool for randomized trials (RoB2) and the Risk of Bias in Non-randomized Studies - of Interventions (ROBINS-I) tools according to the design of the included studies [31, 32]. Any discrepancies in assessments were resolved through consultation with a third expert author (S.S.) to ensure consistency and accuracy in the evaluation process, and inter-examiner agreement was evaluated using Cohen’s kappa statistic.

### Data synthesis

Qualitative synthesis of the data collected from the included studies was carried out in six categories: bibliographic information, study characteristics, risk of bias, radiation properties, de-epithelialization efficacy, and clinical outcomes. Due to the heterogeneity in outcome measures, qualitative reporting, and the limited number of available studies, quantitative analysis was deemed not possible.

### Certainty of evidence assessment

The certainty of evidence was assessed using a modified version of the Grading of Recommendations Assessment, Development and Evaluation (GRADE) approach, tailored for use in studies where meta-analysis is not conducted and findings are summarized narratively [33]. The assessment considered key domains, including methodological limitations (risk of bias), indirectness, imprecision, inconsistency, and potential publication bias across the body of included studies to determine the overall certainty of evidence.

## Results

### Study selection

**Fig. 1:**
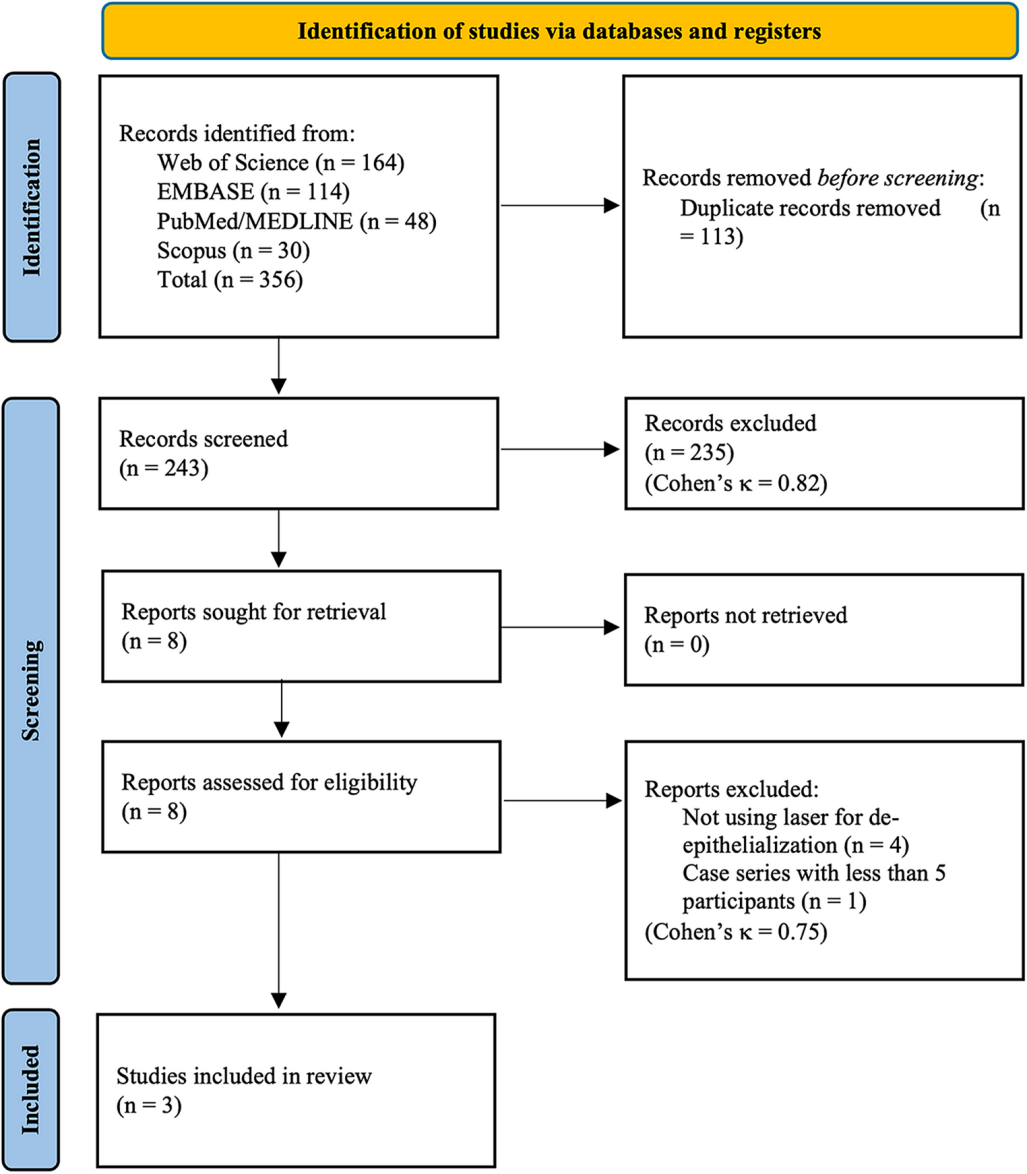
PRISMA flowchart of the review

After combining citations from all databases and eliminating duplicates, 243 records were screened by title and abstract. Eight studies were identified for full-text review, all of which were successfully accessed and retrieved. Among these, three studies met the eligibility criteria and were included in the review, while five studies were excluded (Supplementary Table 4). The inter-examiner agreement was 0.82 (95% CI: 0.66–1) for the title and abstract screening, and 0.75 (95% CI: 0.29–1.00) for the full-text review. Figure 1 presents the PRISMA flow diagram illustrating this process.

### Study characteristics

All of the included studies were published between 2016 and 2020. Two studies were conducted in Turkey [29, 34], while Japan [23] contributed with one study. Except for the study conducted in Japan, both remaining studies took place in academic settings. With regard to the design of the included studies, two were randomized controlled clinical trials (RCTs) [29, 34], one with a parallel-arm design and the other with a split-mouth design, while one study was a single-arm clinical trial [23]. A total of 68 patients were assessed in the included studies, presenting with 105 gingival recession defects. To treat these conditions, 83 FGGs were harvested. Except for one graft, which was taken from the tuberosity [23], all others were harvested from the lateral hard palate. Fifty-two of these FGGs were de-epithelialized using LASERs—26 extra-orally and 26 intra-orally—while the remaining 31 FGGs were de-epithelialized with blades extra-orally and served as controls. Except for six grafts where coronal advancement of soft tissue was achieved using the tunneling technique, the remaining 77 grafts were treated with either trapezoidal or triangular coronally advanced flaps (CAFs). A summary of the characteristics of the included studies is presented in Table 2. Both RCTs reported outcomes based on a 6-month follow-up, whereas the pre-post study provided data from a 1-year follow-up.

**Table 2:** A summary of the methodology and findings of the included studies. (All the values before vs. present LASER group)

Author (s), year	Study design	*N*# of patients	Donor site (#*N*)	De-epithelialization technique (#*N*)	Soft tissue augmentation procedure (#*N*)	De-epithelialization histological outcome	Follow-up	Clinical outcomes	Patient-reported outcomes	Complications
Yoshino et al., 2020 [23]	Single arm clinical trial	11(with 21 gingival recession defects)	Palate (20), Tuberosity (1)	Extra-orally with CO_2_ Laser (21)	• CAF + CTG (15) • Tunneling + CTG (6)	• 0.5 W: More than half of the epithelium was left. • 1 W: The epithelium was completely removed, keeping the morphology of the rete ridge and papillary layer of the lamina propria uninvaded. • 2 W: The entire epithelium and outer surface of lamina propria were removed.	1 year	CRC: 36.3% MRC: 41.0% KTW gain: 2.9 ± 0.3 (***p*** **< 0.001**) RD reduction: 1.85 ± 0.2 (***p*** **< 0.001**)	NR	None.
Gursoy et al., 2019 [34]	Split-mouth randomized controlled clinical trial	5 (with 28 gingival recession defects)	Palate (10)	• Extra-orally with Er: YAG Laser (5) • Extra-orally with 15-C blade (5)	CAF + CTG (10)	NR	6 months	CRC: 82%±4 vs. 86%±36 (*p >* 0.05) MRC: 96.36%±8.09 vs. 97.14 ± 7.26 (*p >* 0.05) KTW gain: 1 ± 0.84 vs. 1.32 ± 1.27 (*p >* 0.05) GT gain: 1.82 ± 0.57 vs. 2.64 ± 0.2 (***p*** **= 0.006**) RD reduction: 2.25 ± 0.93 vs. 2.36 ± 0.63 (*p >* 0.05) CAL gain: 2.53 ± 0.82 vs. 2.36 ± 0.76 (*p >* 0.05) PD reduction: 0.27 ± 0.79 vs. 0.0 ± 0.55 (*p >* 0.05). Color match of the graft was superior in LASER group according to periodontists’ idea (***p*** **= 0.008**)	Color match of the graft was superior in the LASER group (***p*** **= 0.048**)	None.
Ozcelik et al., 2016 [29]	Randomized controlled clinical trial	52 (with 52 isolated gingival recession defects)	Palate (52)	• Intra-orally with diode laser (26) • Extra-orally with 15-C blade (26)	CAF + CTG (52)	NR	6 months	CRC: 84.6% vs. 80.8% (*p >* 0.05). MRC: 96.3% vs. 95.9% (*p >* 0.05). KTW gain: 4.6 ± 1.3 vs. 4.3 ± 1.1 (*p >* 0.05). CAL gain: 4.5 ± 1.6 vs. 4.5 ± 1.3 (*p >* 0.05). PD reduction: 0.0 ± 0.8 vs. 0.2 ± 1.0 (*p >* 0.05). RD reduction: 2.25 ± 0.93 vs. 2.36 ± 0.63 (*p >* 0.05) RW reduction: 2.8 ± 0.7 vs. 2.7 ± 0.8 (*p >* 0.05) ERSA reduction: 13.9 ± 4.5 vs. 13.8 ± 6.3 (*p >* 0.05) Graft thickness: 1.24 ± 0.12 vs. 1.26 ± 0.13 (*p >* 0.05) Surgical chair-time: 34.15 ± 2.29 vs. 39.19 ± 3.60 (***p =*** **0.0001**).	None were totally attributable to de-epithelialization procedure.	None.

Abbreviations: CAF: Coronally Advanced Flap; CTG: Connective Tissue Graft; W: Watt; CRC: Complete Root Coverage, MRC: Mean Root Coverage; KTW: Keratinized Tissue Width; RD: Recession Depth; NR: Not Reported; Er:YAG: Erbium-doped Yttrium Aluminum Garnet; GT: Gingival Thickness; CAL: Clinical attachment level; PD: Probing Depth; RW: Recession Width; ERSA: Exposed Root Surface Area;

### Risk of bias in studies

**Fig. 2:**
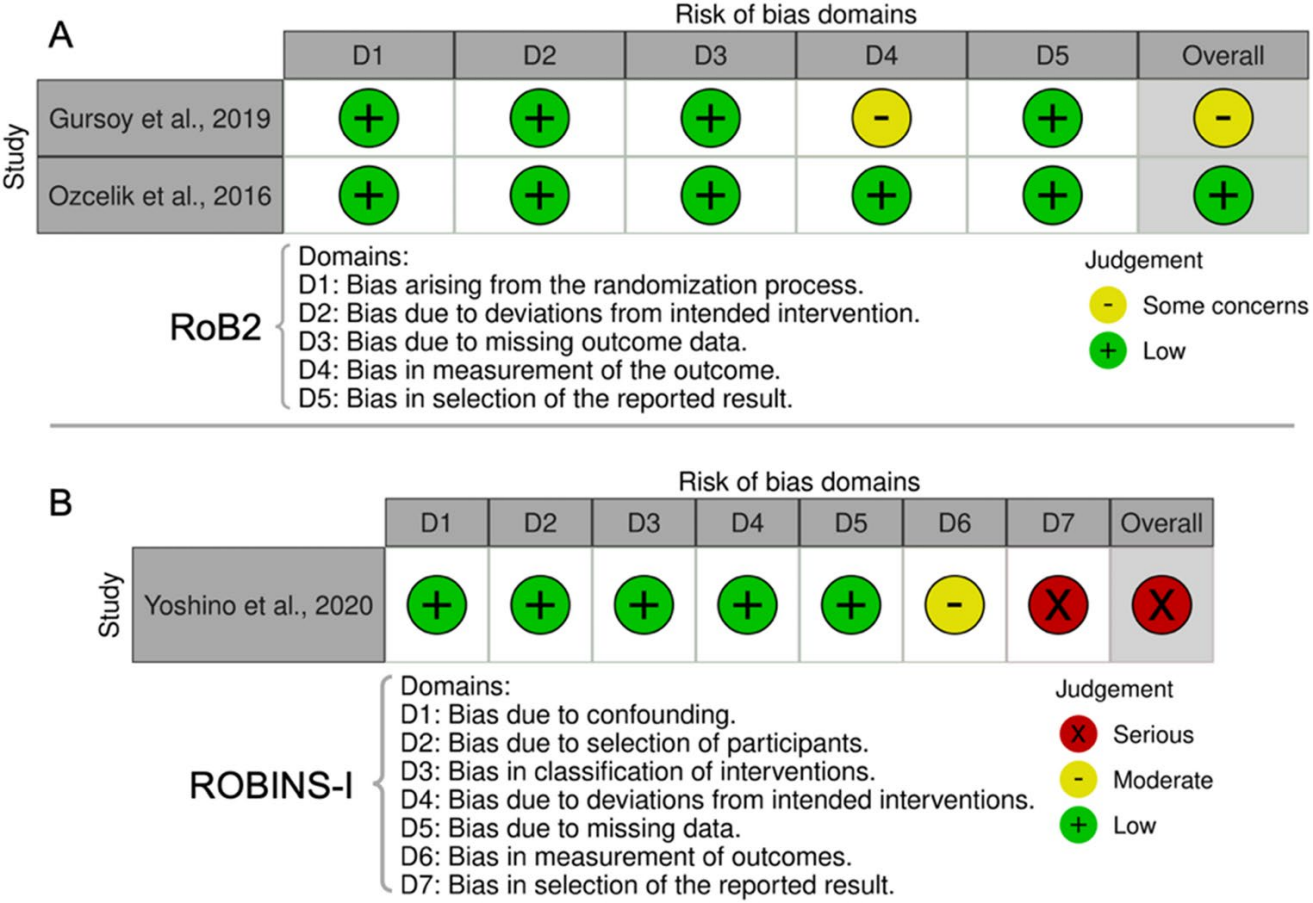
Risk of bias assessment of the included studies according to **(A)** RoB2 and **(B)** ROBINS-I tools

As shown in Fig. 2, the RoB2 tool analysis indicated that one of the included RCTs presented with a low overall risk of bias [29], while the other had some concerns [34]. Also, according to the ROBINS-I tool, the only single-arm clinical trial included in this review had a serious overall risk of bias, mainly due to inappropriate and vague reporting of epithelium removal outcome measures [23]. The inter-examiner agreement was 0.83 (95% CI: 0.51–1).

### Radiation parameters

CO_2_ (10600 nm) [23], Er: YAG (2940 nm) [34], and diode (810 nm) [29] LASERs were utilized in the included studies. The emission mode was pulsed in one studies, with a pulse frequency of 40 Hz and a duration of 200 ms [34], while the remaining two studies used a continuous wave (CW) mode [23, 29]. As shown in Table 3, radiation parameters varied widely across studies, and in many cases, they were either missing or not reported. For example, radiation time, power density (intensity(, energy, and energy density (fluence) were not reported in any of the studies. Two studies utilized 1 W power [23, 29], and 2 W power was used in one study [34]. Spot area was only reported in one study, with a diameter of 0.15 mm [23]. The application method was non-contact in two studies, with distances of 1 mm [34] and 10 mm [23] from the target tissue, while another study used a contact method [29]. Finally, the angulation of radiation was reported only in the study that performed intraoral de-epithelialization, with an angle of 45 degrees [29].

**Table 3:** A summary of radiation parameters of lasers used in the included studies

Author(s), year	Laser type	Parameters										Cooling method
		Wavelength (nm)	Emission Mode (repetition rate, pulse duration)	Diameter of spot area (mm)	Application technique	Angulation of radiation	Total radiation time (s)	Power (W)	Power density (intensity) (W/cm^2^)	Energy (J)	Energy density (fluence) (J/cm^2^)	
Yoshino et al., 2020 [23]	CO_2_	10,600	CW	0.15*	Non-contact (10 mm distance)*	NR	NR	0.5, 1, 2	NR	NR	NR	Air flow*
Gursoy et al., 2019 [34]	Er: YAG	2940*	Pulsed (40 Hz, 200 ms)	NR	Non-contact (1 mm distance)*	NR	NR	2*	NR	NR	NR	Water irrigation
Ozcelik et al., 2016 [29]	Diode (AlGaAs)	810	CW	NR	Contact	45° to the surface	NR	1	NR	NR	NR	NR

Abbreviations: **nm**: nanometer; **mm**: millimeter; **s**: second; **W**: Watt; **cm**: centimeter; **J**: Joule; CW: Continuous Wave; **NR**: Not Reported; **Er**:YAG: Erbium-doped Yttrium Aluminum Garnet; **Hz**: hertz; **ms**: millisecond;

* This value or information was not directly mentioned in the article and was calculated according to other parameters or extracted from the instructions of the device

### De-epithelialization efficacy

Quantitative evaluation of epithelium removal, either percentage of removal or grafts with complete removal, were not reported in any of the included studies; however, Yoshino et al. reported that they performed histological assessment on the remnants of harvested FGGs [23]. They de-epithelialized these remnants with three different powers of 2, 1, and 0.5 W. The specimens were then stained with hematoxylin and eosin and analyzed under a microscope. They found that in the specimen treated with a 2 W power, the entire epithelium and the outer surface of the lamina propria were removed. In contrast, the 0.5 W setting was unsuccessful in removing more than half of the epithelium. Treatment with a power of 1 W resulted in the complete removal of the epithelium while maintaining the morphology of the rete ridges and leaving the papillary layer of the lamina propria intact [23].

### Clinical outcomes

No complications or safety issues were reported in the included studies. Both RCTs found no statistically significant differences between LASER and blade de-epithelialization in CRC, MRC, KTW gain, RD reduction, CAL gain, or probing depth (PD) reduction [29, 34]. However, Gursoy et al. found that while gingival thickness was greater in blade group (1.82 ± 0.57 mm in LASER vs. 2.64 ± 0.2 mm in blade [*p* = 0.006]), the color match of the graft was superior in the LASER group, as assessed by both patients (*p* = 0.048) and periodontists (*p* = 0.008) [34]. Additionally, Ozcelik et al. reported that surgery chair-time was significantly lower in LASER de-epithelialization (34.15 ± 2.29 min in LASER vs. 39.19 ± 3.60 min in blade [*p* = 0.0001]) [29]. Conversely, Ozcelik et al. found no statistically significant difference in recession width (RW) reduction, exposed root surface area reduction, and graft thickness between LASER and blade de-epithelialization [29]. In the pre-post study by Yoshino et al., significant reduction in RD (1.85 ± 0.2 (*p* < 0.001)) and increase in KTW (2.9 ± 0.3 (*p* < 0.001)) were observed following treatment of gingival recession defects with LASER de-epithelialized CTGs [23]. They reported MRC and CRC rates of 41.0% and 36.3%, respectively [23]. Patient-reported outcome measures were reported in both included RCTs [29, 34]. Gursoy et al. reported that, based on patient scores, graft color match was significantly better in the LASER group (*p* = 0.048), while contour, consistency, contiguity, and keloid formation showed no differences between LASER and blade de-epithelialization [34]. Nevertheless, since photobiomodulation therapy was applied to the palatal wound in the study assessing post-operative pain and morbidity using the Oral Health-Related Quality of Life (OHQoL) and Visual Analogue Scale (VAS) for discomfort, isolating the true effect of LASER de-epithelialization on these parameters in this study was not possible [29].

### Certainty of evidence

As shown in Table 4, the quality of evidence was rated as very low, primarily due to methodological limitations—particularly risk of bias and study design—as well as imprecision related to small sample sizes and publication bias.

**Table 4:** Quality of evidence according to the GRADE framework modified for studies without meta-analysis

Certainty assessment						Effect	Number of participants (studies)	Certainty in the evidence
Study design	Methodological limitations of the studies	Indirectness	Imprecision	Inconsistency	Likelihood of publication bas			
Randomized and non-randomized trials	Serious	Not serious	Very serious	Not serious	Serious	All studies showed better or similar results to blade de-epithelialization	83 (3)	⨁◯◯◯ Very low

## Discussion

In this systematic review, a comprehensive literature search was conducted to assess the effectiveness of LASER for de-epithelializing FGGs and its clinical outcomes in soft tissue augmentation procedures. A total of three prospective clinical studies—two RCTs and one pre-post study—were included, all of which primarily compared the clinical outcomes of gingival recession treatment using LASER-de-epithelialized versus blade-de-epithelialized CTGs. The scarcity of available studies on this topic limited the feasibility of conducting any quantitative synthesis, including meta-analyses. However, a qualitative analysis of the included studies suggests that LASERs may serve as a reliable and efficient alternative to conventional blade de-epithelialization. Except for gingival thickness, which was greater with blade de-epithelialization than with LASER in one RCT [34], all other outcome variables—including MRC, CRC, KTW gain, RD reduction, RW reduction, CAL gain, and PD reduction—were comparable when blade- or LASER-de-epithelialized CTGs were used to treat gingival recession defects. Moreover, using LASER for de-epithelialization resulted in improved outcomes in terms of graft color match and reduced surgery time [29, 34]. Considering that Gursoy et al. did not report graft thickness and used a 2 W power setting, which has been shown to remove both the outer lamina propria and the entire epithelium [23], the lower gingival thickness they observed may be attributed to a reduced graft thickness.

The only study evaluating epithelium removal lacked quantitative measures and did not report the number or size of epithelial remnants [23]. Similarly, Gursoy et al. provided a comparative histological view of Er: YAG laser and blade-treated sites without detailed analysis [34]. As a result, the certainty of evidence regarding the appropriate dosing and effectiveness of CO₂ LASER for de-epithelialization remains very low, highlighting the need for further research.

Two studies assessed patient reported outcomes. Gursoy et al. found that patients perceived better esthetic results with LASER-de-epithelialized CTGs [34]. They attributed the favorable results of the laser-de-epithelialized site to the uniform application of the Er: YAG laser on the graft, which resulted in complete removal of the epithelium and, consequently, a more seamless integration with the CAF [34]. Moreover, this more aesthetically favorable outcome may be partially attributed to the smaller increase in gingival thickness at the grafted sites. Nevertheless, the patient-reported outcomes in Ozcelik et al.’s study primarily focused on post-operative pain and discomfort, assessed using the Oral Health-Related Quality of Life (OHQoL) and VAS for discomfort [29]. Since this study applied photobiomodulation therapy to the palatal wound, assessing the effect size of de-epithelialization methods on these outcomes was not feasible.

It was also reported by Ozcelik et al. that surgery time was significantly shorter using LASER de-epithelialization technique [29]. Even though the total chair-side time of the surgery reported in the original study proposing this technique is less than what is reported in Ozcelik et al.’s study (35.8 ± 3.4 min in original study vs. 39.19 ± 3.6 min), Zucchelli’s extra-oral de-epithelialization via a blade is generally considered a very technique-sensitive method. In contrast, de-epithelialization using a LASER, when performed with appropriate radiation parameters, follows a clear and predictable protocol that many clinicians may find faster and more convenient. However, none of the studies included in this systematic review reported clinician satisfaction with the procedure.

The only study using LASER for de-epithelialization that was excluded from this review was a case series by Lin et al. [35]. In this study, an Er, Cr: YSGG LASER with a wavelength of 2780 nm was used intra-orally to de-epithelialize a total number of four FGGs: one for treating a patient with multiple adjacent gingival recessions, one for a patient with implant soft tissue dehiscence, and two for a patient with alveolar ridge soft tissue deficiency [35]. All cases in this study were successfully treated using a power of 2.5 W, which exceeds the power levels used in any of the studies included in this review. In Lin et al.’s study, the LASER probe was positioned in light contact with the target tissue at a 90-degree angle [35]. They state that the main advantage of LASER-assisted de-epithelialization of FGG is that it is performed intraorally before harvesting and allows for uniform application in a controlled manner. In their study, they presented histological sections showing complete epithelium removal [35]. However, similar to Yoshino et al.’ study, Lin et al. merely stated that the epithelium was completely removed without providing any quantitative analysis [23, 35]. Additionally, the histological sections indicate that de-epithelialization extended significantly beyond the epithelial layer, resulting in the removal of part of the lamina propria.

Laser-assisted epithelium removal, extensively studied in ophthalmology, functions through two primary mechanisms: photothermal ablation and photodisruption. In photothermal ablation, epithelial tissue absorbs laser energy, resulting in a rapid temperature rise that leads to vaporization and tissue removal. Photodisruption, on the other hand, utilizes high-intensity laser pulses to ionize the target tissue, generating plasma and acoustic shock waves that break down cellular structures. These mechanisms allow for precise and controlled epithelial removal across various medical applications, reducing damage to surrounding tissues and improving patient outcomes [36, 37].

The primary limitation of this study was the high scarcity of available reports of LASER de-epithelialization in the literature. Moreover, only one study in this review demonstrated a low overall risk of bias [29], indicating that most included studies did not fully adhere to the principles of rigorous clinical trials. Furthermore, no study has explicitly evaluated the efficacy of epithelium removal with LASER using highly reliable methods, such as those employed by Couso-Queiruga when comparing intra- and extra-oral de-epithelialization with a blade [19]. This leaves the question of how effectively LASERs can remove epithelium from FGGs unanswered. These shortcomings highlight the need for well-designed, high-quality histological studies to validate the hypothesis of LASER’s efficacy in de-epithelialization. Such studies would lay the groundwork for conducting RCTs to determine optimal irradiation parameters.

Investigations regarding possible adverse effects of de-epithelialization with LASER on the graft have been reported in the literature. However, in most of these studies, the terms *ablation* and *incision* are used interchangeably. In an ex vivo study, Kawamura et al. evaluated porcine gingival tissue changes following ablation with different LASERs and electro scalpels [38]. Among the LASERs tested, CO₂ caused the highest surface temperature during irradiation (over 500 °C), followed by Diode, Nd: YAG, Er: YAG, and Er, Cr: YSGG, in decreasing order. CO₂ and Diode LASERs led to severe carbonization, while Nd: YAG caused moderate effects, and Er: YAG and Er, Cr: YSGG showed minimal to slight carbonization [38]. Scanning electron microscopy (SEM) revealed smooth surfaces with Er: YAG and Er, Cr: YSGG, while the others produced rough textures. Histological analysis showed that Er: YAG resulted in the thinnest thermally affected area (38 μm). Overall, they concluded that Er: YAG and Er, Cr: YSGG caused the least surface alteration, and water spray significantly reduced thermal destructive changes [38]. In periodontal practice, in addition to de-epithelialization of grafts, LASERs have also been employed to de-epithelialize periodontal flaps. In a review study by Rossmann and Israel, they presented evidence from animal studies indicating that the underlying connective tissue is minimally affected by LASER ablation and that healing is not impaired or delayed [39]. Conversely, Centty et al. utilized a CO₂ LASER with a power of 8 watts to de-epithelialize gingival flaps and found that the underlying connective tissue is composed of three layers: a necrotic zone with coagulated collagen and almost no cells; a thermal effect zone; and a zone of unaffected connective tissue [40]. In the thermal effect zone, collagen appeared glassy, and the surrounding tissue showed a mix of normal and homogenized collagen fibers. This area also showed coagulated and blocked small blood vessels (arteries, arterioles, and veins) and moderate inflammation, mainly involving plasma cells [40]. Further research is required to precisely address the question of how the structure of connective tissue and the viability of its cells are influenced by LASER de-epithelialization.

To the best of the authors’ knowledge, no other reports on LASER de-epithelialization of FGG exist in the literature beyond those included in this review. Also, no review studies on this topic are available in the literature for comparison with the findings of this study.

In summary, while LASER shows promise for de-epithelializing FGGs, further research is needed to establish optimal treatment parameters and uncover its true efficiency in removing the epithelium. Developing standardized and effective irradiation protocols will require additional studies that thoroughly document irradiation parameters and investigate the nuances of dosage and application methods.

## Conclusions

This systematic review summarized the existing evidence on the use of LASER for de-epithelializing FGGs. The primary challenges in drawing robust quantitative conclusions and establishing protocols for future large-scale, high-quality clinical trials include the limited number of available studies and inconsistencies in reporting procedural details and outcomes. Our qualitative analysis suggests that the use of LASER, under specific parameters, may be a reliable alternative to a blade for de-epithelialization, considering MRC, CRC, KTW gain, RD reduction, PD reduction, and CAL reduction as treatment goals. Additionally, LASER-de-epithelialized CTGs may achieve better graft color matching, which is crucial for aesthetic outcomes, while also reducing surgery time. Nonetheless, evidence on the efficiency of LASER for epithelium removal remains limited, highlighting the need for future research to standardize irradiation protocols, improve the reporting of irradiation parameters, expand patient-reported outcomes of the procedure, and quantitatively assess successful de-epithelialization.

## Electronic supplementary material

Below is the link to the electronic supplementary material.

Supplementary Material 1

## Data Availability

No datasets were generated or analysed during the current study.
